# Food Intake and Food Selection Following Physical Relocation: A Scoping Review

**DOI:** 10.3389/phrs.2023.1605516

**Published:** 2023-02-01

**Authors:** Trevor Kouritzin, John C. Spence, Karen Lee

**Affiliations:** ^1^ Division of Preventive Medicine, Department of Medicine, Faculty of Medicine & Dentistry, University of Alberta, Edmonton, AB, Canada; ^2^ Faculty of Kinesiology, Sport, and Recreation, University of Alberta, Edmonton, AB, Canada

**Keywords:** food intake, food selection, fruit and vegetable consumption, physical relocation, neighbourhood food environment

## Abstract

**Objectives:** To synthesize the current available evidence on the changes in food intake and food selection after physical relocation in non-refugee populations.

**Methods:** The inclusion criteria were studies with a measurement of food selection and/or food intake in non-refugee populations where physical relocation had occurred with self-reported or objective assessment of the neighbourhood physical environment before and after relocation. Databases searched included MEDLINE, EMBASE, CINAHL and SCOPUS from 1946 to August 2022.

**Results:** A total of four articles met the inclusion criteria. Overall, these studies gave longitudinal (*n* = 2) and cross-sectional (*n* = 2) evidence to suggest that moving to an urban neighbourhood with more convenience stores, cafés and restaurants around the home was associated with an increase in unhealthy food intake in adult populations. Additional factors such as income, vehicle access, cost, availability and perceptions of the local food environment played a role in shaping food selection and food intake.

**Conclusion:** Four internal migration studies were found. The limited evidence base calls for more research. Future studies should include children and apply appropriate research designs to account for neighbourhood self-selection and concurrent life events. International migration studies should include assessment of neighbourhood physical environments pre- and post-relocation.

## Introduction

Unhealthy diets are a significant risk factor for chronic disease, disability and premature death [1]. One of every five deaths across the globe is attributable to suboptimal diet [2]. Key drivers of unhealthy eating include increased consumption of processed foods high in calories, salt, sugar and saturated fat, and a lack of whole grains, nuts, seeds, legumes, fruits and vegetables [3]*.* Evidence-based elements of a healthy diet include emphasizing fruits and vegetables, unsaturated fats, whole grains, plant protein sources and limiting consumption of trans and saturated fats, highly refined grains and sugary beverages [4]. A key healthy dietary factor in many available guidelines is fruit and vegetable consumption. The 2015 to 2020 Dietary Guidelines for Americans recommend at least 2½ servings of vegetables and 2 servings of fruit per day [5]. The World Health Organization [6] and the World Cancer Research Fund [7] recommend 5 servings of fruit and vegetables per day. Various reviews have associated low intake of fruits and vegetables with cardiovascular diseases, hypertension, hypercholesterolemia, osteoporosis, many cancers, chronic obstructive pulmonary diseases, respiratory problems and poor mental health [8–11]. A meta-analysis of 16 cohort studies following 469,551 participants provided evidence that a higher consumption of fruit and vegetables is associated with a lower risk of all-cause mortality, particularly cardiovascular mortality [12]. Fruit and vegetable intake and selection are thus used as key outcome measurements of healthy eating in this scoping review.

Available data globally suggests insufficient fruit and vegetable consumption. For instance, a majority of adults in Australia, Canada, the UK, and US do not meet recommended fruit and vegetable consumption guidelines [13–15]. *In Sub-*Saharan Africa, daily fruit and vegetable intake (268 g) remain below the World Health Organization’s recommendation (400 g) [16]. Healthcare costs associated with not meeting food guidelines and/or treating obesity range from USD$3.3 billion to USD$50.4 billion in developed countries [13–15, 17]. Similarly, according to the Global Medical Trends Survey [18], healthcare costs attributable to poor dietary habits are projected to rise steadily in Sub-Saharan Africa. The high prevalence of unhealthy eating habits and the economic burden of not meeting dietary recommendations for health suggests that investments in promoting healthy eating have the potential of substantial savings in direct and indirect healthcare costs.

Dietary behaviors and food consumption are shaped by interrelated personal and environmental factors, including knowledge [19], affordability [20], physical neighbourhood environments and accessibility [21]. The complex interplay of personal, cultural and environmental factors impacting dietary behaviors can be categorized and described using the five levels of influence conceptualized by the socio-ecological model (intrapersonal, interpersonal, institutional, community and public policy) [22, 23]. Longitudinal studies linking changes in the local food environment to changes in eating behaviour and diet selection provide evidence that increased numbers of fast food outlets and convenience stores around the home may contribute to a lower diet quality, increased unhealthy food intake and higher BMI [24–27]. Cross sectional studies link availability and accessibility of healthful food sources to healthier dietary patterns, such as increased fruit and vegetable consumption [28]. Therefore, creating neighbourhoods that provide opportunities to purchase healthy food and limit exposure to unhealthy food represents a potential strategy to address some of the contributing factors to the burden of chronic diseases caused by poor dietary intake [29].

Understanding how individuals interact with their physical environment is one crucial component for public health strategies aimed at improving dietary intakes. Previous research usually examines dietary habits and/or neighborhood environment separately, and few studies have dealt with them simultaneously [30]. Residential location refers to the structures in which people live, and the grounds on which such structures are located including, but not limited to, houses, apartments, condominiums and the amenities around them [31]. The distinction between the physical aspects of the environment and its underlying food behaviour influences is also not always clear because people may self-select their residential locations based on multiple, and usually unmeasured, economic and social variables. For example, activity-conscious individuals may be more likely to move to neighborhoods with higher walkability and more recreational facilities [32]. In addition, the wide range of conceptualization of the environment makes it challenging to compare results across studies. Examining the impact of physical relocation (i.e., moving to another neighborhood) may be an efficient way to determine the role of the neighbourhood environment on health. Specifically, analyzing health outcomes following physical relocation represents a different type of natural experiment that allows researchers to compare proximity and access to elements within food environments as a measure of influence.

To date, reviews that examined dietary outcomes following physical relocation have generally been limited to mass migrations such as refugee crises [33–36]. These reviews have found that food insecurity is a marked consequence of international migration and constitutes an emerging global public health problem. Less is understood about the impact of residential relocation on food consumption when moving from neighbourhood to neighbourhood within non-refugee populations. A scoping review aims to map the existing literature in a field of interest in terms of the volume, nature, and characteristics of the primary research [37]. This scoping review synthesized the current evidence on the association between food intake and food selection, and physical relocation in non-refugee populations, where the food environment before and after relocation are also assessed. All studies that had a measurement of food selection and/or food intake after physical relocation (either prospectively or retrospectively) with self-reported or objective assessment of neighbourhood physical environment before and after relocation were included. Non-refugee was defined as an individual who had undergone immigration, migration or relocation due to reasons besides persecution. Food selection was defined by the British Nutrition Foundation definition: the selection of foods for consumption which results from the competing, reinforcing and interacting influences of a variety of factors [38]. Food intake was defined as the daily eating patterns of an individual, including specific foods, calories consumed and relative quantities [39]. Physical relocation was defined as the action of moving to a new place and establishing one’s home there [40]. The research questions included: What studies have been done on food selection and food intake following physical relocation? How does physical relocation affect food selection and food intake? How does physical relocation affect healthy eating outcomes (defined by fruit and vegetable intake)? What is known about the facilitators and barriers to healthy eating (defined by fruit and vegetable intake) following physical relocation?

## Methods

A scoping review provides an overview of the literature on a topic and can be most useful when there is a variety of research designs or an expected scarcity of evidence [41]. Guided by the [37] methodological framework for scoping reviews and recommendations for strengthening methodological rigor, a systematic methodological approach for searching, selecting, summarizing, and synthesizing the existing literature on food intake and food selection following physical relocation in a non-refugee study population was employed.

### Protocol and Registration

Our protocol was drafted using the Preferred Reporting Items for Systematic reviews and Meta-Analyses extension for Scoping Reviews (PRISMA-ScR) [42]. The final protocol was registered prospectively with the Open Science Framework (https://osf.io/3wvfu/) [43].

### Approach

Searches were conducted from the earliest database inception (1946) to August 2022 in the electronic databases MEDLINE, EMBASE, CINAHL, and SCOPUS for peer-reviewed papers. Search terms included key words related to physical relocation, food selection and food intake (see Supplementary Appendix Table SA1). The detailed search strategy for each database is available in the (Supplementarty Appendix). The listed databases were searched and resulting citations were downloaded into Covidence [44].

### Selection Process

The focus of this scoping review was available academic literature. Peer-reviewed studies that had any outcome measurement of food selection and/or food intake where physical relocation had occurred (either prospectively or retrospectively) with self-reported or objective assessment of neighbourhood physical environment before and after relocation were included. Non-English publications, gray literature, studies using only refugees or immigrants as the study population, and/or relocation with limited food intake self-selection were excluded. The last exclusion criterion was chosen because eating behaviours within institutional food environments with minimal dietary self-selection may not be comparable to behaviours determined by availability of choices in neighbourhood food environments.

Following a standard protocol, potential included studies were screened for eligibility based on the title, abstract and full text. Uncertainty was discussed among all authors and any disagreement was resolved by consensus. A PRISMA flow diagram presents the summary of the study selection process (Supplementary Appendix Figure SA1).

### Methodological Quality Appraisal

We did not appraise methodological quality or risk of bias of the included articles, which is consistent with guidance on scoping review conduct [45]. However, characteristics of available studies were extracted and documented to provide information on strengths and weaknesses.

### Data Extraction and Analysis

Publication characteristics, study characteristics and participant information were extracted. Publication characteristics included author, year of publication, publication type, and country in which the study was conducted. Study characteristics included study design, aim and objectives of the study, research methods, neighbourhood environmental attributes (perceived or objectively measured), results and main conclusions. Participant information included number of participants, age and gender. Finally, results regarding sociability and perceived safety were also extracted because both these attributes have also been linked to how the neighbourhood environment could impact residents’ willingness and ability to access nearby food amenities [46]. Sociability was defined as the web of social relationships that surround an individual and the extent to which an individual is connected with others [47]. Safety was defined as how safe individuals feel in their neighborhoods [48].

## Results

### Article Characteristics

The literature search identified 144 potential studies after removing duplicates. A total of 129 irrelevant documents were removed during phase one screening for the wrong outcomes or incorrect study population. For example, many studies focused on displacement of natural disaster victims or cardiometabolic outcomes. Of the 15 full-text studies assessed for eligibility, four studies met the inclusion criteria and were included in the review [49–52]. The first published study of food intake and food selection following physical relocation in non-refugee populations appeared in 2004 [50], followed by 2007(52), 2018 [51] and 2020 [49]. In the studies by Butler et al. (2004) and Papadki et al. (2007), participants relocated out of the family home to attend college; in the study by [51] participants relocated from a rural to urban environment; and in the study by [49] participants relocated from an established neighbourhood to a new residential development. All studies relied on quantitative data and involved adult populations. The study by Butler et al. (2004) used only female participants; the other three studies included both female and male participants. The studies by Butler et al. (2004) and Papadaki et al. (2007) used a cross-sectional study design; the studies by [49] and [51] used a longitudinal study design. Sample sizes ranged from 54 (Butler et al. 2004) to 9,417 [51] participants. Butler et al. (2004) evaluated food intake and food selection 5 months post-relocation, Papadaki et al. (2007) evaluated food intake and food selection 3–4 years post-relocation, [49] used a pre- and 1–2 years post-relocation measurement and [51] used a pre- and 4 years post-relocation measurement. All studies assessed food frequency and included a measurement of food selection and food intake. In addition, the study by [51] included a food diversity score and the study by Butler et al. (2004) included measurements of nutrient self-efficacy and macronutrient consumption. Supplementary Appendix Table SA2 summarizes the characteristics of the included studies. Supplementary Appendix Table SA3 summarizes the results of the included studies.

### Food Selection and Food Intake

Fruit, vegetable, bread/pasta, milk, meat and refined sugar consumption were the most commonly used food intake outcomes. Grocery stores, home meals and meals consumed outside of the home at convenience stores, cafés and restaurants were the most commonly used locations of food selection. A variety of measures were employed to operationalize these concepts including self-administered questionnaires about usual food intake, lifestyle behaviours, perceptions, self-efficacy, socio-demographic variables and measurements of the neighbourhood environmental attributes.

Some studies reported small positive outcomes after relocation (e.g., decreased white bread consumption [52]; a greater percentage of healthy food outlets around the home following relocation was found to be associated with an increase in fruit and vegetable intake [49]). However, the negative effect on food intake and food selection after physical relocation was the more prominent theme. For example, although Papadaki et al. (2007) reported some positive outcomes, students who relocated within Greece when starting university modified their dietary habits in a generally undesirable direction (decreased fresh fruit, raw and cooked vegetables, pulses, seafood, olive oil consumption and increased sugar consumption). Butler et al. (2004) reported a significant increase in alcohol consumed of freshman female college after relocation from home. [49] reported that moving to a new residential development with more convenience stores, cafés and restaurants around the home was associated with an increase in unhealthy food intake. Although researchers reported that a greater percentage of healthy food outlets around the home following relocation was significantly associated with an increase in fruit and vegetable intake, 64% of participants experienced a decline in the percentage of healthy food outlets around the home following residential relocation compared to 25% who experienced an improvement. [51] reported relocating to urban areas resulted in a significant decrease in maize and cassava consumption, and a significant increase in bread, pasta, cereal products, sugar, sweet, pastries, sodas, tea, coffee and meals/snacks consumed outside the house.

As a whole, healthy food intake declined among relocated residents. However, relocation seemed to have a positive impact on sociability as shown by an increase in leisure activities and meals consumed outside the home, especially when residents relocated for university [50, 52] or from a rural to urban environment [51]. There was no change in perceived neighbourhood safety.

### Facilitators and Barriers to Healthy Eating

The most commonly reported facilitators of healthy eating were increased income and food selection from rural migration. Major barriers to healthy eating included lack of time and competing priorities, lack of accessible transportation, no grocery stores within walking distance (as defined by a 1.6-km road network buffer), cost and not adjusting habits to favour a healthier diet.

Papadaki et al. (2007) reported that lack of experience in planning meals, a general lack of interest in food, or lack of time were also barriers for healthier dietary choices and precipitating factors for increased consumption of take-away and convenience meals. [49] reported that having children <18 years of age at home at baseline was associated with an increase in unhealthy food intake, access to a vehicle at baseline was associated with an increase in diet quality and fruit/vegetable intake following relocation, and higher socioeconomic status and increasing hours of work per week was associated with a decrease in unhealthy food intake. The latter is contrary to what was expected as working >40 h per week is associated with time-related barriers to healthful eating in previous literature in adults [53, 54] and young adults [53]. Butler et al. (2004) reported that nutrition self-efficacy, defined by one’s belief in his or her ability to manage a diet even in the face of obstacles such as stress or exposure to unhealthy foods [55], did not change during the first semester of university after physical relocation from home. [51] reported income as the main mediator through which rural-urban migration affected dietary change. If it were not for the increases in income associated with rural-urban migration, there would have be no significant change in consumption. Not surprisingly, the most significant change in consumption was away from traditional staples (maize, cassava) which are typically consumed from one’s own production in rural areas.

## Discussion

This review found a scarcity of literature on residential relocation and food selection and intake in non-refugee and institutional residential populations that included assessments of food environments pre- and post-relocation, with four publications in four countries (three high-income countries and one lower-income country) meeting the inclusion criteria. The small number of studies and heterogenous designs make it difficult to draw conclusions about associations. Overall, these studies provided longitudinal (*n* = 2) and cross-sectional (*n* = 2) evidence to suggest that moving to an urban neighbourhood with more convenience stores, cafés and restaurants around the home was associated with an increase in unhealthy food intake. There is evidence that having a greater percentage of healthy food outlets around the home following relocation was significantly associated with an increase in fruit and vegetable intake; however, a majority of participants experienced a decline in the percentage of healthy food outlets around the home following residential relocation compared to a minority who experienced an improvement. Intrapersonal (individual) level characteristics of food intake included preferences/perceptions and knowledge/skills; interpersonal level characteristics of food intake included food availability, social support, time constraints and culture; community/institution level characteristics of food intake included food availability (stores), school/workplace food environment, eating out and access; and policy level characteristics of food intake included food pricing. Biological and psychological determinants of food selection were not tested in these studies. Other economic, physical and social determinants of food selection included cost, income, availability, skills (e.g. cooking), time, culture, family, peers and meal patterns. Furthermore, factors such as vehicle access and availability of public transportation played a role in shaping food selection and food intake, improving outcomes when present.

In all studies, dietary selection and intake as well as personal context changed significantly following residential relocation. In the Butler et al. (2004) and [52] studies, participants moved out of the family home to a university campus. [52] reported that the majority (73%) of students living away from home lived alone during their studies, 18% shared a flat with friends and a small proportion (8.1%) lived in shared student residences. A finding of interest in the [52] study is that there were no major differences in dietary habits at baseline when students lived in the family home, regardless of whether students came from Athens or other parts of Greece. Both Butler et al. (2004) and [52] found that young adults who relocated when starting university significantly increased convenience and take-away meal consumption. The findings of [52] suggest that food shopping plays a significant role in the forming dietary habits because students still living with their families, where food shopping and cooking were usually performed by a family member, did not change their diets in a major way after starting university. [51] found that compared to household members who remained in their original rural villages, those relocating to urban areas experienced a pronounced shift away from traditional staples and towards more convenience meals away from home. These findings suggest that the ratio of unhealthy to healthy food outlets influences people’s dietary choices, a finding consistent with the previous cross-sectional research exploring the effects of relative and absolute measures of exposure [56, 57]. For example, having a higher number of convenience stores within 3 km [24, 26] and fast food restaurants within 1 km around the home [27] is associated with lower dietary quality in the US. In Canada, individuals living in neighbourhoods with a moderate or high density of fast-food chain restaurants are more likely to be excessive fast-food consumers [58]. In the [49] study, participants moved from a previously established neighbourhood to a new residential development. The new developments were typically located in suburban greenfield areas and infill locations. The majority of participants (64.0% vs. 24.8%) experienced a decline in the percentage of healthy food outlets around the home following residential relocation to a new development. These findings are consistent with previous research that identified an overall lack of healthy food outlets in new developments: 2.3 times more takeaway/fast food outlets than supermarket/greengrocers in new developments compared with 1.7 times in established neighbourhoods [59].

Findings from these studies generally show less healthy food consumption following relocation. This is consistent with previous literature: a systematic review of 11 studies with university students reported higher salt, fat, and added sugar consumption on campus [60]; and empirical evidence shows rural residents tend to have lower calorie intakes and higher dietary quality than their urban counterparts [61]. With the exception [49], where the change was non-significant, all studies reported a significant decrease in fruit and vegetable consumption following physical relocation. While it is difficult to compare the magnitude of effects across studies given the variety of measurements used, previous studies that used survey measures of the food environment consistently reported small but meaningful differences in fruit and vegetable consumption within the different dimensions of “food selection” (biological, economic, physical, social, and psychological determinants) [62]. For example, individuals who reported shopping at a supermarket consumed, on average, 1.22 more servings per day of fruits and vegetables than those who did not [63], and individuals who reported easy supermarket access consumed, on average, 86 more grams per day of fruit (approximately half a serving) than those who reported poorer access [64].

The changes observed in food intakes after relocating are likely also influenced by specific individual factors modifying the way participants respond to a changing environment. For example, having children at home and lower socioeconomic status at baseline were associated with an increase in unhealthy food intake after relocating [49]. Thus, families with children and people living on low incomes may be especially vulnerable to purchasing less healthy convenience foods from accessible food outlets around the home. Previous research also suggests that low-income residents may be more susceptible to unhealthy food intake in environments where there is a high prevalence of unhealthy food outlets [24, 26]. In Edmonton, Canada, the odds of exposure to fast food outlets are greater in areas with more Indigenous peoples, renters, lone parents, low-income households and public transportation commuters [65, 66]. In the [49] study, access to a vehicle at baseline was associated with an increase in diet quality and fruit/vegetable intake following relocation. This suggests that people with vehicles may be better able to travel beyond their immediate neighbourhood to obtain healthy food, increasing their potential to access healthy food. Urban migration may also explain some of the deterioration of fruit and vegetable consumption as individuals are purportedly further from fresh, seasonal local produce.

Residential relocation had some influence on participant behaviour and perceptions. Bvioltsis et al. (2020) found that individual positive perceptions of the local food environment on average decreased from pre-to post-relocation, as indicated by 40.1% of participants reporting a decrease in the presence of a supermarket/grocery store within 15-min walk of home. Previous research has revealed that both objective and perceived measures of increased distance to the nearest supermarket with a good variety of fresh and processed vegetables is associated with decreased daily consumption of fruit and vegetables [67]. Only [51] assessed whether physical relocation affects men and women differently. The changes in the consumption and selection of different food categories after relocation appeared to be similar, except for meals and snacks consumed away from home. The more pronounced increase in the latter food category was driven by male migrants. A potential explanation lies is that women in Africa may often migrate for marriage [68]. Conversely, male migrants are more likely to be unmarried and to live alone, and perhaps are less likely to cook or have someone else preparing food at home.

### Strengths and Limitations

Limitations inherent to scoping review methodology are that they identify available research and point to research that needs to be conducted on a topic rather than contributing essential research. This review is also limited by restricting studies to English-language publications, exclusion of gray literature, and heterogeneous measurement and outcome measures. Furthermore, no intervention studies were identified. Therefore, only observational studies were available and two of the four were cross-sectional further preventing conclusions about causality.

Daily food selection and consumption were estimated through self-reported surveys in all studies, which is subject to measurement error from incorrect recording of food intake and potential reluctance to report consumption of unhealthy foods. For instance, previous literature shows that up to 50% of participants may incorrectly self-report food consumption [69]. There are also challenges separating out what changes in dietary behaviour might be from the move itself rather than the change in residential context. For example, some relocations are associated with negative life events (e.g., divorce, ill health, loss of unemployment). Finally, summarising across diverse environmental attributes and different outcome measurements is methodologically challenging. While categorizing these measures provides a “big picture” of the overall evidence, it may fail to address potential biases in interpretation.

Despite these limitations, this review has multiple strengths including a comprehensive search strategy to identify available evidence on the topic and avenues for further research. The diversity of the geographical locations provides a representation of the changes in food intake and selection following physical relocation in four different continents compared to earlier reviews on the local food environment that have been limited to studies from primarily higher- or upper-middle-income countries [70, 71].

### Conclusion

Residential relocation provides a unique opportunity for studying possible environment-induced changes in food intake and food selection, especially when the environments pre- and post-relocation are assessed. This scoping review identified four studies from three high-income countries and one low-income country: two studies with residential relocation out of the family home to a college campus, one study with residential relocation from a rural to urban environment, and one study with residential relocation from an established neighbourhood to a new residential development. Moving to a new residential development with more convenience stores and restaurants around the home was associated with an increase in unhealthy food intake. Conversely, having a greater percentage of healthy food outlets around the home following relocation was significantly associated with an increase in fruit and vegetable intake; however, a majority of participants experienced a decline in the percentage of healthy food outlets around the home following residential relocation compared to a minority who experienced an improvement. Commonly reported barriers to healthy eating also included lack of time and competing priorities, lack of accessible transportation, no grocery stores within walking distance, cost and not adjusting habits to favour a healthier diet.

The limited evidence base calls for more research examining food intake, food selection and residential relocation that include assessments of food environments pre- and post-relocation. None of the studies looked at residential relocation to a different state/province, which may lead to greater changes in the neighbourhood food environment and more significant dietary changes than relocation within the same state/province. None of the studies included children, who may have different dietary preferences and behavioural influences then adults. Future studies could benefit from using longitudinal and interventional designs, such as quasi-experimental studies and cohort studies [32], evaluating relocation effects over longer follow-up periods, and applying appropriate research methods to account for neighbourhood self-selection and concurrent life events. Additional data from geographically diverse areas, particularly from low-income and middle-income countries, would also add to the current literature.
