# Supplementary material for: Food Intake and Food Selection Following Physical Relocation: A Scoping Review
Source: Public Health Rev. 2023 Feb 1;44:1605516. doi: 10.3389/phrs.2023.1605516 (PMC9928753; doi:10.3389/phrs.2023.1605516)
Supplement: Supplementary file 1 [file DataSheet1.docx]

# **Appendix**

*Figure 1. Preferred Reporting Items for Systematic Reviews and Meta-Analyses (PRISMA) Flow Diagram of Reviewed Research (Edmonton, Canada. 2023).*


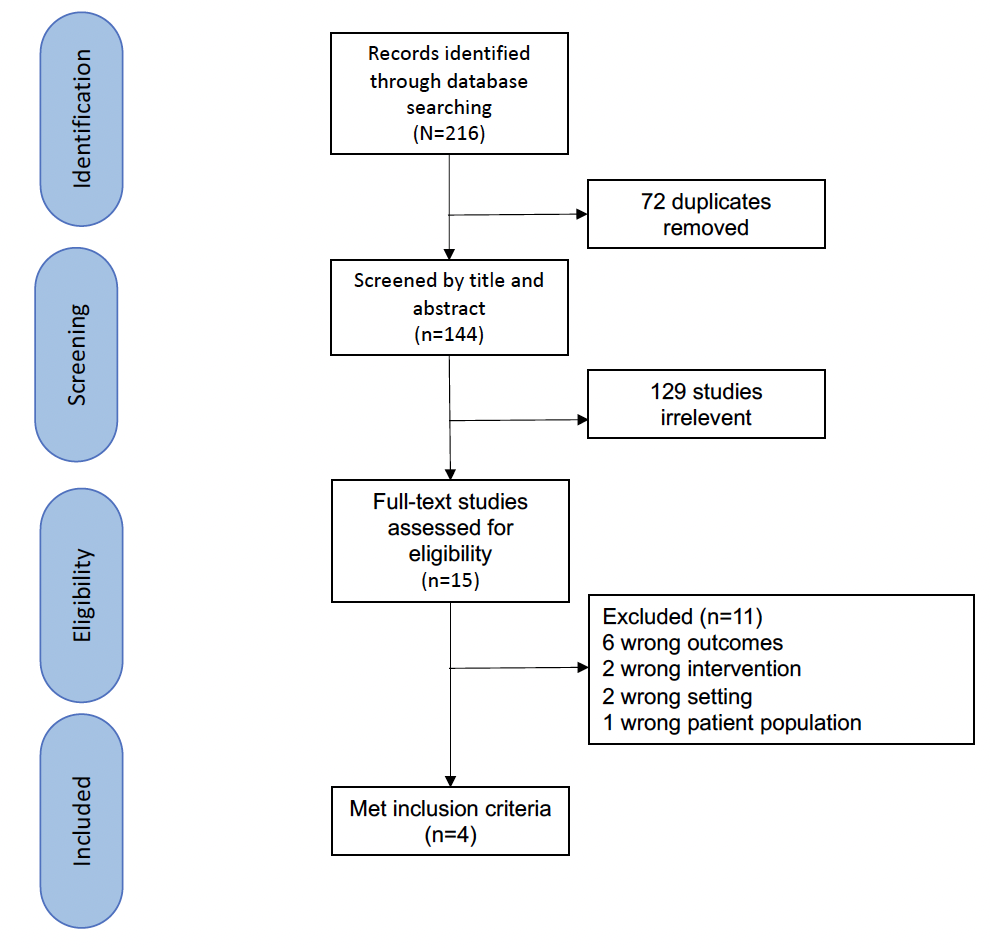


*Table 1. Search Strategy for Article Identification (Edmonton, Canada. 2023).*

| **Concept** | **Search term** |
| --- | --- |
| Physical Relocation | ((physical or residential or residence* or home* or location* or neighborhood* or neighbourhood* or city or cities or urban or rural or town*) adj5 relocat*) |
| Food Intake and Food Selection | exp Feeding Behavior/  exp Food/ or exp "Diet, Food, and Nutrition"/  exp Nutrition Assessment/  (eat OR eats OR eating OR food* OR calorie* OR meal* OR fruit consum* OR vegetable* consum* OR beverage consum* OR water consum* OR diet* behavior?r* OR diet* qualit* OR diet* intak* OR diet* pattern* OR diet* habit* OR malnutr* OR nutrition* OR feeding behavior?r*) |

*Table 2. Characteristics of Included Studies (n=4) (Edmonton, Canada. 2023).*

| **Characteristic** | **Number of articles** | **Percent** |
| --- | --- | --- |
| Geographical region | | |
| Greece | 1 | 25% |
| Australia | 1 | 25% |
| USA | 1 | 25% |
| Tanzania | 1 | 25% |
| Study design | | |
| Cross-sectional | 2 | 50% |
| Longitudinal | 2 | 50% |
| Outcomes (multiple aims possible) | | |
| Food intake | 4 | 100% |
| Food selection | 4 | 100% |
| Food diversity score | 1 | 25% |
| Calories/Macronutrients | 1 | 25% |
| Outcome measurement methods (multiple measurements possible) | | |
| Questionnaire | 4 | 100% |

*Table 3. Summary of Results of Included Studies (Scoping Review, low- and high- income countries, 2004-2020, n=4) (Edmonton, Canada. 2023).*

| **Author, Year, Publication Type, Country** | **Study Design** | **Aims and Objectives of The Study** | **Research Methods** | **Study Population** | **Neighbourhood Environmental Attributes** | **Intervention Results** | **Safety & Sociability** | **Conclusions and Additional Comments** |
| --- | --- | --- | --- | --- | --- | --- | --- | --- |
| Papadaki et al, 2007, Journal, Greece | Cross-sectional | · Assess the effect of relocating away from, or staying in the family home on the dietary habits of Greek  undergraduate university students. | · Self-administered questionnaire about consumption of selected foods, general food habits and demographic characteristics. | · 84 under graduate students (61.9% female) aged 22.3 $\pm$ 1.8 years.  *· Findings suggests that food shopping plays a significant role in forming dietary habits; students still living at home where food shopping and cooking were usually performed by a family member did not change their diets in a major way since starting university.* | · Urban migration may also explain some of the deterioration from the traditional Greek diet as individuals are purportedly further from fresh, seasonal local produce. | · Diets of university students living at home did not change after starting university.  · Students who relocated when starting university modified their dietary habits in a generally undesirable direction; (decreased fresh fruit, raw and cooked vegetables, pulses, oily fish and seafood, olive oil consumption and increased sugar consumption). But, also small positives (decreased white bread and full fat Greek yogurt consumption). | · Greece has a strong  cultural identity, with food playing an important role in  everyday activities, religious festivals, family and social  events. Family influence may have made students still living at home  be less susceptible to change their dietary habits. | · Findings suggest the importance of the family environment in forming dietary habits.  · Neither group of students achieved recommend intakes, with students who lived at home eating on average 5.2 servings of fruit and 8.3 servings of vegetables per week, while students who lived away from home consuming on average 4.7 servings of fruit and 4.3 servings of vegetables per week. |
| Butler et al, 2004, Journal, USA | Longitudinal | · Change in diet and body weight among freshman female college students during the first semester of university after relocation from home. | · Block food frequency questionnaire and Sallis exercise and nutrition self-efficacy questionnaire. | · 54 female subjects. 8.54% were 17 years old. 91.64% were 18 years old. |  | · Pre-relocation caloric intake of 2205/day.  · Post-relocation caloric intake of 1857/day.  · Post-relocation significant decrease in vegetables, bread/pasta, milk and meat food groups.  · Post-relocation significant increase in alcohol consumed. | · Self-efficacy questionnaire suggests the stability of students' confidence to overcome diet obstacles because student perceptions about self-efficacy did not change over time.  *· Significant decreases in total physical, work, and sport activities,*  *· Significant increase in leisure activities; primarily walking to and from classes.* | · Deficiencies in both pre- and post- relocation intake of daily vegetables, fruits, breads and pasta, and meats.  · Participants, however, seem to be consuming adequate amounts of milk at both time periods. |
| Bivoltsis et al, 2020, journal, Australia | Cross-sectional | · To examine the associations of changes in the local food environment, individual behaviours and perceptions, and changes in dietary intake, following relocation from an established neighbourhood to a new residential development. | · Participants completed a self-reported questionnaire on health, lifestyle behaviours, perceptions, usual food intake and socio-demographic variables prior to relocating and 1–2-years post move. | · 1200 participants (38.3% male) aged 40.5 $\pm$ 11.8 years at baseline. | · New developments were typically located in outer suburban, greenfield areas and infill locations; further from the Perth Central Business District.  · Compared with previous established neighbourhoods, the food environments within the new developments were characterised by a lower percentage of healthy food outlets and greater distances from home to the nearest grocery store. | · Moving to a new residential development with more convenience stores, cafés and restaurants around the home was associated with an increase in unhealthy food intake. But, was partially mediated by individual behaviours and perceptions.  · A greater percentage of healthy food outlets around the home following relocation was associated with an increase in fruit/vegetable intake. | · Increasing hours of work per week (and income) at baseline was associated with a decrease in unhealthy diet after relocation.  · Participants with children < 18 years at home before moving had a significant increase in unhealthy diet following relocation. | · Increased  spatial exposure to convenience stores, cafés restaurants  increased unhealthy food intake, whilst an increased  percentage of healthy food outlets around the home  increased healthy food intake. |
| Cockx et al, 2018, journal, *Tanzania* | Longitudinal | · To compare dietary patterns of individuals before and after relocation from rural to urban areas. | · One-week food consumption recall questionnaire at the household level, combined with a questionnaire on eating away from home at the individual level. | · Baseline: 16,058 individuals from 3,284 households.  · Post relocation: 9,417 individuals living in 2580 households.  · 913 individuals migrated during the 4-year study window (56% female); 710 moved to another rural area and 238 moved to an urban area. | · Although currently a low-income and low human development, Tanzania is one of the world’s most rapidly growing and urbanizing countries; average annual urban population growth accounting to over 5% and average annual GDP per capita growth rate close to 3%.  · Tanzania is also characterized by large internal migration movements.  *· Compared to rural, urban environments contain a proliferation of small*  *mini-markets and clustered food shops.* | · Relocating to urban areas caused a significant decrease in maize, cassava consumption and significant increase in bread, pasta, cereal products, sugar, sweet, pastries, sodas, tea, coffee, and meals/snacks consumed outside the house.  · Moving to an urban area did not appear to contribute to higher intake of more nutritious food groups. | · Increased restaurant meals and food consumed away from home in urban relocated participants. | · Rural to urban migration men ate significantly more meals/snacks consumed outside of the house than women.  · Average controlled direct effect (a measure of the role of a mediator in causal mechanism) of rural-urban migration indicated that income is an important mediator through which rural-urban migration effects dietary change. |

***Medline Search Strategy***

1. ((physical or residential or residence* or home* or location* or neighborhood* or neighbourhood* or city or cities or urban or rural or town*) adj5 relocat*).mp.

2. exp Feeding Behavior/

3. exp Food/ or exp "Diet, Food, and Nutrition"/

4. exp Nutrition Assessment/

5. (eat or eats or eating or food* or calorie* or meal* or fruit consum* or vegetable* consum* or beverage consum* or water consum* or diet* behavio?r* or diet* qualit* or diet* intake* or diet* pattern* or diet* habit* or malnutr* or nutrition* or feeding behavio?r*).mp.

6. 2 or 3 or 4 or 5

7. 1 and 6

Results = 36

## ***Embase Search Strategy***

1 ((physical or residential or residence* or home* or location* or neighborhood* or neighbourhood* or city or cities or urban or rural or town*) adj5 relocat*).mp.

2 exp feeding behavior/

3 exp nutritional assessment/

4 exp food/

5 (eat or eats or eating or food* or calorie* or meal* or fruit consum* or vegetable* consum* or beverage consum* or water consum* or diet* behavio?r* or diet* qualit* or diet* intake* or diet* pattern* or diet* habit* or malnutr* or nutrition* or feeding behavio?r*).mp.

6 2 or 3 or 4 or 5

7 1 and 6

Results = 54

## ***Cinahl Search Strategy***

S1 ((physical or residential or residence* or home* or location* or neighborhood* or neighbourhood* or city or cities or urban or rural or town*) N5 relocat*)

S2 (MH "Eating Behavior+")

S3 (MH "Food") OR (MH "Food Preferences") OR (MH "Food Habits") OR (MH "Health Food+") OR (MH "Food Quality+") OR (MH "Food Intake+") OR (MH "Food Deserts") OR (MH "Food and Beverages+") OR (MH "Dietary Carbohydrates+") OR (MH "Dietary Fats+") OR (MH "Dietary Proteins+") OR (MH "Fruit+") OR (MH "Meals+") OR (MH "Meat+") OR (MH "Nutrients+") OR (MH "Raw Foods") OR (MH "Salads") OR (MH "Vegetables+") OR (MH "Meat Substitutes") OR (MH "Soy Foods+") OR (MH "Seafood+") OR (MH "Snacks") OR (MH "Seeds+") OR (MH "Nuts+") OR (MH "Mushroom, Edible") OR (MH "Infant Food+") OR (MH "Honey") OR (MH "Herbs, Seasoning") OR (MH "Functional Food") OR (MH "Food, Fortified") OR (MH "Fast Foods") OR (MH "Dairy Products+")

S4 (MH "Nutritional Assessment")

S5 (eat or eats or eating or food* or calorie* or meal* or “fruit consum*” or “vegetable* consum*” or “beverage consum*” or water consum* or “diet* behavio?r*” or “diet* qualit*” or “diet* intake*” or “diet* pattern*” or “diet* habit*” or malnutr* or nutrition* or “feeding behavio?r*”)

S6 S2 OR S3 OR S4 OR S5

S7 S1 AND S6

Results = 15

## ***Scopus Search Strategy***

TITLE-ABS-KEY((physical OR residential OR residence* OR home* OR location* OR neighborhood* OR neighbourhood* OR city OR cities OR urban OR rural OR town*) W/5 relocat*) AND TITLE-ABS-KEY(eat OR eats OR eating OR food* OR calorie* OR meal* OR "fruit consum*" OR "vegetable* consum*" OR "beverage consum*" OR "water consum*" OR "diet* behavio?r*" OR "diet* qualit*" OR "diet* intake*" OR "diet* pattern*" OR "diet* habit*" OR malnutr* OR nutrition* OR "feeding behavio?r*" )

Results = 110
